# Largely shared neural codes for biological and nonbiological observed movements but not for executed actions in monkey premotor areas

**DOI:** 10.1152/jn.00296.2021

**Published:** 2021-08-11

**Authors:** Davide Albertini, Marco Lanzilotto, Monica Maranesi, Luca Bonini

**Affiliations:** ^1^Department of Medicine and Surgery, University of Parma, Parma, Italy; ^2^Department of Psychology, University of Turin, Torino, Italy

**Keywords:** dorsal premotor cortex, mesial premotor cortex, mirror neurons, population dynamics, neural subspace

## Abstract

The neural processing of others’ observed actions recruits a large network of brain regions (the action observation network; AON) in which frontal motor areas are thought to play a crucial role. As the discovery of mirror neurons (MNs) in the ventral premotor cortex, it has been assumed that their activation was conditional upon the presentation of biological rather than nonbiological motion stimuli, supporting a form of direct visuomotor matching. Nonetheless, nonbiological observed movements have rarely been used as control stimuli to evaluate visual specificity, thereby leaving the issue of similarity among neural codes for executed actions and biological or nonbiological observed movements unresolved. Here, we addressed this issue by recording from two nodes of the AON that are attracting increasing interest, namely, the ventrorostral part of the dorsal premotor area F2 and the mesial presupplementary motor area F6 of macaques while they *1*) executed a reaching-grasping task, *2*) observed an experimenter performing the task, and *3*) observed a nonbiological effector moving in the same context. Our findings revealed stronger neuronal responses to the observation of biological than nonbiological movement, but biological and nonbiological visual stimuli produced highly similar neural dynamics and relied on largely shared neural codes, which in turn remarkably differed from those associated with executed actions. These results indicate that, in highly familiar contexts, visuomotor remapping processes in premotor areas hosting MNs are more complex and flexible than predicted by a direct visuomotor matching hypothesis.

**NEW & NOTEWORTHY** Pioneering studies on mirror neurons (MNs) in premotor areas emphasized the absence of response to the sight of nonbiological moving objects, suggesting a match between execution and observation activities. This study shows that although premotor neurons can discriminate between biological and nonbiological observed movements, these visual stimuli rely on largely shared neural codes, which differ strongly from those associated with executed actions.

## Graphical abstract

**Figure:**
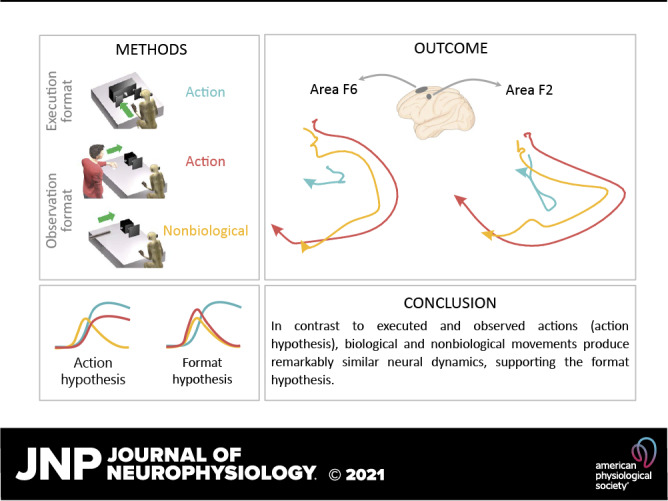

## INTRODUCTION

Since the pioneering study on mirror neurons (MNs), a class of cells originally discovered in area F5 of the macaque discharging during both action execution and observation, it was reported that “responses to tools or to objects moved in such a way as to imitate the effective action were usually weak or absent altogether” (1). These findings emphasized the similarity between the neural codes for observed and executed actions, that is, the visual and motor formats. Nonetheless, subsequent studies have shown that MNs in various nodes of the action observation network (AON) (2) can exhibit visual responses to actions performed with tools (3, 4), actions implied by moving cues (5–10), or even withheld actions signaled by an instructive cue (11, 12), suggesting a greater flexibility and broader relationship between the visual and motor codes.

**Figure 1.**
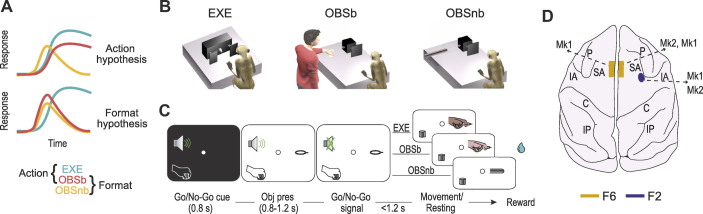
Experimental hypotheses, behavioral tasks, and recorded regions. *A*: the action hypothesis predicts greater similarity between EXE and OBSb than between either and OBSnb; the format hypothesis predicts greater similarity between OBSb and OBSnb than between either and EXE. *B*: schematic representation of the setting for each task: execution (EXE), observation of a biological (OBSb), or nonbiological (OBSnb) movement in the monkey’s extrapersonal space. *C*: temporal sequence of task events. Following the presentation of a central fixation point (17), fixation onset caused the presentation of a cue sound, either 1,200 or 300 Hz, which instructed the monkey to grasp (Go cue) or refrain from grasping (No-Go cue) the subsequently presented object, respectively. Following object presentation (Obj pres) the sound ceased and the monkey reached, grasped, and pulled (for 0.8 s) the object (or in the No-Go condition, remained still for 1.2 s) to receive a fixed amount of juice reward, automatically delivered after each correctly performed trial. In the No-Go trials of OBSb the monkey had to simply observe the experimenter remaining still until the end of the trial. *D*: recorded regions in the two monkeys reported on the schematic reconstruction of monkey Mk2’s brain. C, central sulcus; IA, inferior arcuate sulcus; IP, intraparietal sulcus; P, principal sulcus; SA, superior arcuate sulcus.

Recent studies have shown that the population dynamics associated with action execution and observation exhibit similarities in area F5 but not in M1 (13, 14), but they have not tested nonbiological motion stimuli. Here, we recorded from two important nodes of the AON—the ventrorostral part of the dorsal premotor area F2 (15) and the mesial presupplementary motor area F6 (16)—under two main alternative hypotheses: *1*) an “action hypothesis” (Fig. 1*A*), which predicts greater similarity in the representation of “actions,” regardless of their visual or motor format, and *2*) a “format hypothesis” (Fig. 1*A*), which predicts greater similarity between the visual formats, regardless of the biological or nonbiological nature of the stimuli.

## METHODS

### Animal Models

Two purpose-bred, socially housed male macaques (Mk1, *Macaca nemestrina*, 9 kg, and Mk2, *Macaca mulatta*, 7 kg) were used for the present study. Monkeys were first prepared for electrophysiological recordings, as previously described (18) and trained to perform the tasks described in the *Apparatus and Behavioral Paradigm* section. All experimental protocols complied with the European Law on the Protection of Animals Used for Scientific Purposes (2010/63/EU), were approved by the Veterinarian Animal Care and Use Committee of the University of Parma (Prot. 78/12, 17/07/2012 and Prot. 91/OPBA/2015), and were authorized by the Italian Ministry of Health (D.M. 294/2012-C, 11/12/2012 and 48/2016-PR, 20/01/2016).

### Apparatus and Behavioral Paradigm

Monkeys performed a reaching-grasping Go/No-Go task (Fig. 1*B*, EXE) with three different objects (a ring, a small cone, and a big cone) as potential targets, to be grasped with three different grip types (hook grip, side grip, and whole hand prehension, respectively). Furthermore, they observed the same task performed by an experimenter in their extrapersonal space (Fig. 1*B*, OBSb) (19, 20) and a variant of the observation task (Fig. 1*B*, OBSnb) in which an elongated object (a metal cylinder) was moved with nonbiological kinematics along the trajectory followed by the experimenter’s arm during OBSb (only the ring object in the Go trials was used in this task). The cylinder’s movement was triggered by a light smack applied on its extremity (invisible to the monkey) by the experimenter, which activated an automatic drawer-like sliding mechanism producing a regular and perfectly linear (hence nonbiological) shift forward of the cylinder toward the target, where it stopped. Trials were separated by a variable (from 1 to 1.5 s) intertrial period. The temporal sequence of task events was the same in the three tasks (EXE, OBSb, and OBSnb, Fig. 1*C*), run in different blocks.

The task phases were automatically controlled by LabView-based software: error trials were discarded (no reward was delivered) and repeated until at least 10 trials were collected for each condition. Here, we compare Go trials of EXE, OBSb, and OBSnb, in addition to the No-Go trials of OBSb as a control (hereafter referred to as NOGO task).

### Recording Techniques

Area F2 (Fig. 1*D*) was studied with acutely inserted linear silicon probes (21, 22) with 16 recording sites spaced by 250 μm apart along 3.75 mm of an 8-mm shank (80 μm wide × 100 μm thick). Area F6 (Fig. 1*D*) was investigated with four chronically implanted multishaft three-dimensional (3-D) arrays of linear silicon probes with eight recording sites per shaft and two parallel modules of four shafts per probe (64 channels per probe). Previous studies have presented the reconstruction of the location of the recording sites (19, 23). The signal was amplified and sampled at 40 kHz with a 16-channel Omniplex recording system (Plexon). In the case of the chronic implants, different sets of 16 channels were recorded only once during separate sessions on different days.

Monkey had to maintain central fixation throughout the entire trial duration in all tasks, which was monitored with an eye-tracking system (20; the tolerance radius of the fixation window was set at 5°). Previous analyses of the electromyographic activity of proximal and distal forelimb muscles of these two monkeys during the tasks (20) allow us to exclude the possibility that preparatory motor activity is present during the No-Go and observation trials.

### Analysis of Neural Activity

Spike sorting was performed off-line with fully automated software (24). Uniform and restrictive criteria were applied to the selection of single units in both areas (25) and nonseparable spikes were also considered as multiunit activity (altogether referred to as “units”). Units were preliminarily tested by comparing their baseline activity (500 ms before object presentation) with each bin in the interval from 600 ms before to 600 ms after movement onset (one-tailed sliding *t* test, window = 200 ms, step = 20 ms, *P* < 0.05, uncorrected). We regarded as facilitated or suppressed all those neurons with at least five consecutive bins significantly greater or lower than baseline activity, respectively. Neurons that did not meet this criterion were considered unmodulated. Only units modulated during OBSb and/or OBSnb were included in subsequent analyses.

#### Population analysis.

We computed the object-averaged net soft-normalized activity for all selected units and tasks (25) and used it to produce the heatmaps. In this study, we were not interested in the contextual effect of the cue sound, which has been described in previous studies (20, 25), therefore, we focused our analysis on the time period following object presentation, taking as baseline the 500-ms interval before this event. We grouped units into facilitated or suppressed, depending on the sign of their average modulation during the movement period within each task (±600 ms relative to movement onset in EXE, OBSb, and OBSnb; ±600 ms relative to the No-Go signal in OBSb). To test whether neurons were significantly facilitated/suppressed, we used the 10 trials with the ring object as a target, which was identical for all tasks.

A one-way repeated-measures ANOVA was applied to the net soft-normalized activity (averaged in a movement epoch corresponding to the 500-ms interval after movement onset) taking the task as main factor (four levels: EXE, OBSb, OBSnb, and NOGO); a Tukey–Kramer test was used for post hoc comparisons. The fraction of units exhibiting a significantly different modulation between OBSb and OBSnb was obtained by applying a two-tailed paired *t* test between the net soft-normalized activity of the ring-object trials of the two tasks (averaged in the 500-ms interval following movement onset, α = 0.05).

#### Neural subspace analysis.

We quantified the similarity in the neural population codes among tasks by computing the residual variance obtained after projecting the neural trajectory of one task onto the neural subspace of another (26). We first calculated the soft-normalized firing rates of each unit in the time interval −600/+800 ms around movement onset for each task (or in the same interval around the No-Go signal in the NOGO task). Specifically, the spiking activity of each neuron was binned in 20-ms time windows, trial averaged, smoothed with a 60-ms Gaussian kernel, and soft-normalized by its absolute maximum (across conditions and tasks, +5 spk/s). We thus obtained 10 matrices, each of dimensions *T* x *N* (*T* being the number of time bins, *N* the number of units), as follows: one matrix for each object tested in EXE, OBSb, and NOGO, and one for the unique object used for OBSnb.

First, for any pair of tasks A and B, we applied principal component analysis (PCA) to the corresponding mean-centered data matrices *X_A_* and *X_B_* (two principal components typically captured >60% of the total variance), obtaining the coefficients of the first two principal components *V_A_* and *V_B_*. Next, we evaluated the overlap or “alignment” of task *A* over task *B* by projecting the neural activity of *A* onto the principal components *V_B_* and computing the residual variance normalized by the variance captured by the first two principal components *V_A_*, as follows (13):

$$alignment_{AB}=\frac{tr(V_{B}^{T}cov\left(X_{A}\right)V_{B})}{tr(V_{A}^{T}cov\left(X_{A}\right)V_{A})}.$$

The alignment ranges from 0 (if neural subspaces are orthogonal) to 1 (if neural subspaces are perfectly aligned). As an estimate of the between-tasks alignment, we took the average alignment across every pair of objects of two different tasks; similarly, to estimate the within-task alignment, we took the average alignment across every pair of objects within that task. Then, to quantify the difference between alignments, we randomly sampled with replacement from the considered population a number of units equal to the population size, and we calculated for that sampled population the between- and within-task alignments and the pairwise alignment differences among them. We repeated this procedure 2,000 times and considered any two alignments significantly different from each other if their alignment difference was higher or lower than 0 (corresponding to a two-tails α = 0.05) in at least 97.5% of the iterations.

The drawings of neural population trajectories were produced as follows. Given the neural activity matrices *X* of a given population (obtained as described above), we normalized each matrix for the number of units *N* contributing to it; this allowed us to visually compare the amplitude of neural trajectories of populations with a different number of units. Then, we normalized the resulting matrices for the square root of their corresponding total variances to make the amplitude of the projected population trajectories comparable across tasks (irrespective of the magnitude of their overall modulations), thereby reflecting the value of alignment. Finally, the resulting activity matrices were projected onto the first two PCs of EXE and OBSb (which explained the largest fraction of variance of object-averaged EXE and OBSb activity, respectively), and the resulting two-dimensional neural trajectories were averaged across objects to obtain a single trajectory for each task.

## RESULTS

We tested 549 units (175 single units and 374 multiunits) from area F6 (*N* = 357; 178 from Mk1 and 179 from Mk2) and F2 (*N* = 192; 27 from Mk1 and 165 from Mk2) with all tasks. Of these, 155 became active only during EXE (*N* = 134) or were not significantly activated in any of the tasks (*N* = 21). The remaining 394 units (*N* = 164 from Mk1 and *N* = 230 from Mk2) were significantly modulated during OBSb and/or OBSnb; of these, 267 were recorded from area F6 and 127 from area F2: these units constituted the data set of the present study.

**Figure 2.**
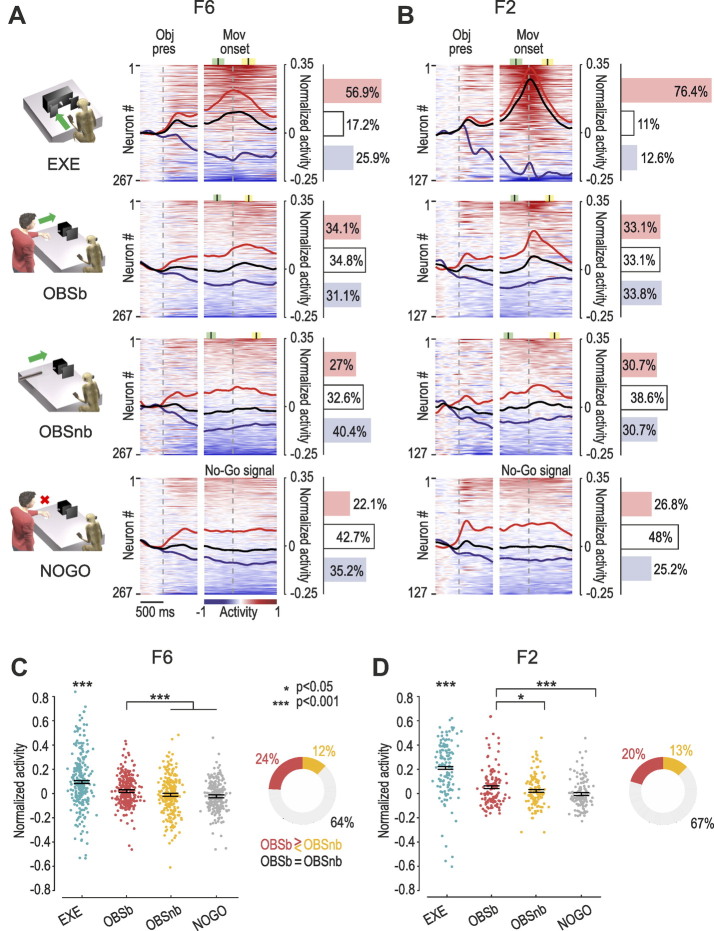
Neural-activity time course in the different tasks. *A*: heat maps of neural activity in area F6 during each task. Each line represents one unit. Units are ordered (from *top* to *bottom*) based on the magnitude of their activity with respect to baseline (red = facilitated, blue = suppressed) in the interval −600/+600 ms relative to movement (Mov) onset (or in the case of NOGO, the No-Go signal), independently for each task. Black lines represent the averaged response of each population as a whole, whereas red and blue lines represent the averaged response of units with overall positive or negative modulation in the movement epoch, respectively (see methods). The histograms on the right indicate the percentage of significantly facilitated (red), suppressed (blue), and nonsignificant (white) units (*P* < 0.05, see methods) in each area. Colored markers represent the average ±1 standard deviation of the Go/No-Go signal (green) and pulling onset (yellow). *B*: heat maps of neural activity in area F2. Conventions as in *A*. *C*, *left*: average net soft-normalized activity of F6 units during the overt movement epoch (500-ms after movement onset) of EXE, OBSb, OBSnb, and NOGO. Each dot represents one unit. One-way repeated-measures ANOVA (*F*_3,798_ = 43.51, *P* = 4.8·10^−26^, η^2^ = 0.083) followed by Tukey–Kramer post hoc test indicates higher activity in EXE relative to all the other tasks and in OBSb relative to OBSnb and NOGO. *Right*: percentage of units with preference for OBSb vs. OBSnb (χ^2^ = 11.23, *P* = 8.1·10^−4^). *D*: same as in *C* for F2 units. *Left*: one-way repeated-measures ANOVA (*F*_3,378_ = 49.27, *P*= 6.7·10^−27^, η^2^ = 0.200) followed by Tukey–Kramer post hoc test indicates higher activity in EXE relative to all the other tasks and in OBSb relative to OBSnb and NOGO. *Right*: percentage of units with preference for OBSb vs. OBSnb (χ^2^ = 2.38, *P* = 0.123). EXE, execution; Obj Pres, object presentation; OBSb and OBSnb, observation of a biological or nonbiological movement, respectively, in the monkey’s extrapersonal space.

During EXE, the majority of the recorded units in both F6 (56.9%, Fig. 2*A*) and F2 (76.4%, Fig. 2*B*) exhibited facilitated responses, in contrast to the observation tasks, in which the modulation of activity was lower and characterized by a more balanced number of facilitated and suppressed units (Fig. 2, *A* and *B*, OBSb and OBSnb). During the No-Go condition of OBSb (NOGO), the unmodulated units prevailed (42.7%), in line with the essentially motor nature of the two areas. Figure 2, *C* and *D* shows that, although most of the units in both F6 (64%) and F2 (67%) showed no significantly different activation between OBSb and OBSnb, the average modulation in both areas was stronger for OBSb than for OBSnb and NOGO, and units with a preference for OBSb were more numerous than those preferring OBSnb, especially in F6.

**Figure 3.**
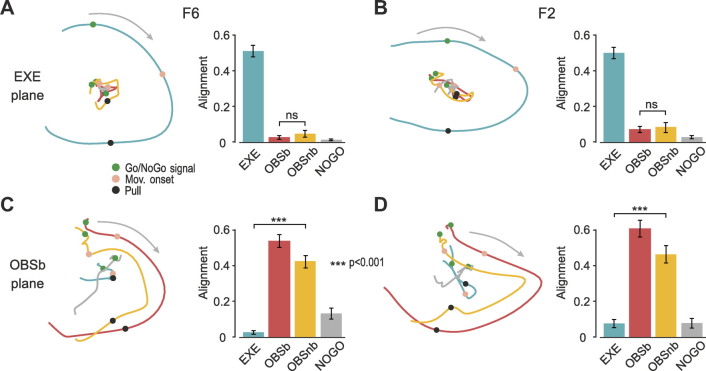
Neural similarity of population dynamics among tasks. *A*: *left*: projections of F6 neural population trajectories (trials and objects averaged) of different tasks onto the neural plane defined with reference to EXE (explained variance: PC_1_ = 33.5%, PC_2_ = 21.2%). *Right*: alignment index among tasks. Error bars were obtained using bootstrap repetitions by resampling units (see methods). EXE showed the highest alignment (*P* ≈ 0 for all comparisons); NOGO showed the lowest (*P* = 0.163 vs. OBSb; *P* = 0.035 vs. OBSnb, which in turn did not differ from each other, *P* = 0.087). *B*: same as *A* for area F2 (explained variance: PC_1_ = 38.7%, PC_2_ = 17.8%). EXE showed the highest alignment (*P* ≈ 0); NOGO showed the lowest (*P* = 0.080 vs. OBSb; *P* = 0.076 vs. OBSnb, which in turn did not differ from each other, *P* = 0.60). *C*: population trajectories and alignment index as in *A*, but with reference to OBSb (explained variance: PC_1_ = 32.1%, PC_2_ = 13.2%). OBSb showed the highest alignment (*P* ≈ 0); for all other comparisons OBSnb > NOGO > EXE (*P* ≈ 0). *D*: same as *C* for area F2 (explained variance: PC_1_ = 28.8%, PC_2_ = 23.7%). OBSb showed the highest alignment (*P* ≈ 0); OBSnb > NOGO and EXE (*P* ≈ 0), which in turn did not differ from each other (*P* = 0.52). Note that the same results are obtained if the analyses are applied in individual monkeys, separately. EXE, execution; Mov. onset, movement onset; NOGO, No-Go signal; OBSb and OBSnb, observation of a biological or nonbiological movement, respectively, in the monkey’s extrapersonal space.

Next, we assessed the similarity in the neural codes of biological and nonbiological movements at the population level by applying recently proposed approaches (13, 27). For each task, we projected the activity during the epoch of interest (i.e., movement/No-Go epoch, depending on the task) onto the plane defined by the first two principal components of EXE for both F6 (Fig. 3*A*) and F2 (Fig. 3*B*), and quantified the fraction of residual variance by computing the alignment index (see methods). We found that the trajectories of OBSb and OBSnb were smaller than that of EXE but, more importantly, that their alignment with the neural plane of EXE did not differ significantly in either F6 (bootstrap procedure *P* = 0.087, see methods) or F2 (*P* = 0.60), indicating that, contrary to the “action hypothesis” (Fig. 1*A*), OBSb did not exhibit greater similarity with EXE than with OBSnb.

The same analysis was performed by taking OBSb as a reference. In both areas (Fig. 3, *C* and *D*), the neural trajectories of OBSb and OBSnb followed a similar evolution, strikingly different from that of EXE and NOGO. Importantly, the alignment of OBSnb with OBSb was significantly greater than that of EXE in both F6 (*P* ≈ 0) and F2 (*P* ≈ 0), in line with the prediction of the “format hypothesis.”

## DISCUSSION

In this study, we contrasted two main alternative hypotheses (Fig. 1*A*): according to the “action hypothesis,” one would expect greater similarity in the representation of “actions,” regardless of their visual or motor format, as compared to nonbiological motion stimuli (1, 28, 29); according to the “format hypothesis,” by contrast, one would expect greater similarity between the visual format of biological and nonbiological stimuli than between either and executed actions (5, 30, 31). Our findings indicate that although some neurons fired more strongly for observed actions than for nonbiological movements, in both areas F6 and F2 the neural representations of biological and nonbiological movements exhibit considerable similarity and strongly differed from the neural representations of executed actions, lending clear support to the format hypothesis.

It should be noted that the very low degree of overlap between the visual and motor codes reported here seems to contrast with the findings of previous studies (13–15). However, some important differences may explain this discrepancy. First, in these studies, the target of the experimenter’s action was closer to the monkey, which is known to exert a considerable effect on premotor neurons’ visual responses (16, 17, 20, 32, 33). Second, exploratory eye movements, which the monkey was allowed to perform in the aforementioned studies but not in our experiment, may have facilitated action observation responses (33), thereby increasing the visuomotor similarity. Nonetheless, even if the distance from the target and the fixation may have caused a reduced similarity between action observation and execution in our experiment, these constraints were the same for both observation tasks and hence cannot account for the remarkable similarity observed between biological and non-biological stimuli. A final possibility is that the discrepancy with the above mentioned previous studies may be due to the fact that they focused on F5 neurons whereas the present study dealt with F2 and F6; the remarkable similarity in the visuomotor properties of F5 and the ventrorostral portion of F2 reported by previous studies (15) makes this interpretation less plausible (at least for F2), and suggest that similar result could be obtained in area F5 as well, in line with previous studies (5).

Future studies with longitudinal chronic recording approaches may address the issue of whether and how premotor neuronal activity becomes so similar for visual stimuli that are remarkably different from the physical and perceptual point of view. One possibility is that the highly predictable task context, in which the monkey was overtrained, had created a generalization between the biological action (which the monkey was used to seeing in that context for a very long time) and the nonbiological stimulus, consistently with the fact that even neurons in the primary motor cortex can become active in similar conditions (8, 9). The findings of previous studies in both ventral (11, 12), dorsal (10), and mesial (20) premotor cortices suggest that visually triggered activity in highly predictable contexts has a considerable anticipatory capacity: the same activity could then be evoked by the sight of similar temporal sequence of events (14) despite perceptually different kinematics features. The hypothesis of an increased neural similarity caused by the repeated association between biological and nonbiological movements along the same spatial trajectory is supported by previous human fMRI data showing that areas of the human MN system can activate similarly for hand movements and meaningless artificial movements of objects in space, likely because an association of objects’ movements with biological movements is evoked (30). An additional, and not mutually exclusive, hypothesis is that greater neural similarity between (visual) formats is the result of the rehearsal of a shared motor representation afforded by both the visual stimuli (34). This interpretation should lead to the hypothesis that, although apparently “visual” in nature, both observed actions and nonbiological motion stimuli produce much greater activation when presented within the monkey’s operational space, especially when from a subjective viewpoint (16), thereby confirming their eminently pragmatic nature.

In summary, our findings show that in two key regions of the monkey AON, biological and nonbiological visual stimuli recruit highly overlapping neural substrates and dynamics, emphasizing the plasticity and the generalization capacities of the visual-to-motor mapping in premotor brain regions, whose functional role still needs to be explored with causal techniques (35).

## DATA AND CODE AVAILABILITY

All data and code are available upon reasonable request. Requests should be made to and will be fulfilled by D.A. (davide.albertini@unipr.it).

## GRANTS

This work was supported by the European Research Council (ERC) under the European Union’s Horizon 2020 Research and Innovation Program (ERC starting Grant No. 678307 to L.B.) and by the FARE program of the Italian Ministry of Education, Universities and Research (MIUR) (Grant No. R16PWSFBPL to L.B.). M.L. is currently supported by an ERC Consolidator Grant 2017 (Grant Agreement 772953).

## DISCLOSURES

No conflicts of interest, financial or otherwise, are declared by the authors.

## AUTHOR CONTRIBUTIONS

M.L., M.M., and L.B. conceived and designed research; M.L., M.M., and L.B. performed experiments; D.A. analyzed data; D.A., M.L., M.M., and L.B. interpreted results of experiments; D.A. prepared figures; D.A. and L.B. drafted manuscript; D.A., M.L., M.M., and L.B. edited and revised manuscript; D.A., M.L., M.M., and L.B. approved final version of manuscript.
